# Synthesis, In Vitro, and In Silico Analysis of the Antioxidative Activity of Dapsone Imine Derivatives †

**DOI:** 10.3390/molecules26195747

**Published:** 2021-09-22

**Authors:** Ricardo Guzmán-Ávila, Mayra Avelar, Edgar A. Márquez, Julio C. Rivera-Leyva, José R. Mora, Virginia Flores-Morales, Jesús Rivera-Islas

**Affiliations:** 1 Laboratorio 4, Facultad de Farmacia, Universidad Autónoma del Estado de Morelos, Av. Universidad 1001, Cuernavaca 62209, Mexico; ricardo.guzman@uaem.edu.mx (R.G.-Á.); julio.rivera@uaem.mx (J.C.R.-L.); 2 Laboratorio de Síntesis Asimétrica y Bioenergética (LSAyB), Ingeniería Química (UACQ), Universidad Autónoma de Zacatecas, Campus XXI Km 6 Carr. Zac-Gdl, Zacatecas 98160, Mexico; mayra.avelar@uaz.edu.mx; 3 Grupo de Investigación en Química y Biología, Departamento de Química y Biología, Universidad del Norte, Km 5 vía Puerto Colombia 1569, Barranquilla Atlántico 081007, Colombia; ebrazon@uninorte.edu.co; 4 Grupo de Química Computacional y Teórica (QCT-USFQ), Departamento de Ingeniería Química, Universidad San Francisco de Quito, Diego de Robles y Vía Interoceánica, Quito 170901, Ecuador; jrmora@usfq.edu.ec

**Keywords:** dapsone imines, dapsone-derivatives, antioxidant in vitro

## Abstract

Dapsone (DDS) is an antibacterial drug with well-known antioxidant properties. However, the antioxidant behavior of its derivatives has not been well explored. In the present work, the antioxidant activity of 10 dapsone derivatives 4-substituted was determined by an evaluation in two in vitro models (DPPH radical scavenging assay and ferric reducing antioxidant power). These imine derivatives **1**–**10** were obtained through condensation between DDS and the corresponding aromatic aldehydes 4-substuited. Three derivatives presented better results than DDS in the determination of DPPH (**2**, **9**, and **10**). Likewise, we have three compounds with better reducing activity than dapsone (**4**, **9**, and **10**). In order to be more insight, the redox process, a conceptual DFT analysis was carried out. Molecular descriptors such as electronic distribution, the total charge accepting/donating capacity (I/A), and the partial charge accepting/donating capacity (ω^+^/ω^−^) were calculated to analyze the relative donor-acceptor capacity through employing a donor acceptor map (DAM). The DFT calculation allowed us to establish a relationship between GAP_HOMO-LUMO_ and DAM with the observed antioxidant effects. According to the results, we concluded that compounds **2** and **3** have the lowest *R_a_* values, representing a good antioxidant behavior observed experimentally in DPPH radical capturing. On the other hand, derivatives **4**, **9**, and **10** display the best reducing capacity activity with the highest ω^−^ and *R_d_* values. Consequently, we propose these compounds as the best antireductants in our DDS imine derivative series.

## 1. Introduction

Dapsone (4,4′-diaminodiphenylsulfone, DDS) is an aniline derivative that belongs to the sulfone drug class. It is mainly used for leprosy treatment in combination with rifam-picin and clofazimine [1,2]. Additionally, DDS has shown a prophylactic effect against *T*. *gondii* and *P. jiroveci* on clinical trials with HIV patients [3,4]. Furthermore, an antiparasitic effect against *P. falciparum* has been reported with DDS in combination with chlorproguanil [5]. Once absorbed, DDS is metabolized into monoacetyldapsone and *N-*hydroxyldapsone [6], being the one oxidation product responsible for the methemoglobinemia [7,8,9].

Moreover, DDS possesses an anti-inflammatory effect similar to other non-steroidal anti-inflammatory drugs [10]. It is a consequence of scavenging for oxidizing species, such as superoxide dismutase, peroxidase, and compounds such as β-carotene [11]. This activity has been explored in neuroscience research, where DDS diminishes the damage caused by oxidative stress in murine models [12].

The antioxidant properties of DDS have been explored as a corrosion inhibitor on steel imbibed on the acid medium (HCl 1 M and H_2_SO_4_ 0.5 M), showing an anticorrosive activity > 90% at DDS 400 ppm [13]. DDS imine derivatives with salicylaldehyde [14], indol-3-carboxaldehyde, thiophene-2-carboxaldehyde [15], and benzaldehyde [16] have shown the anticorrosive effect at the same level as the parent drug.

A structural modification strategy for drugs containing the amine functionality con-sists of derivatizing into amides or imines. The imine group has the advantage, from the synthetic point, of being easily accessible, which can improve the solubility and biological activity compared to the precursor amines [17,18]. Derivatization also positively impacts DDS solubility (0.16 mg/mL) when it reacts with aliphatic amino acids to reach aqueous solubilities higher than 25 mg/mL [19]. Additionally, the derivatives reported by Wadher et al., five derivatives of 4-substituted DDS imines maintain their antibacterial and antifungal activity against *E. coli*, *S. aureus*, *A. niger*, and *C. albicans* [20]. In recent years, the use of computational methods has increased considerably since they allow us to understand and explain various structural and electronic properties of molecules and correlate them with experimental data [21]. The calculation of quantum molecular descriptors is a useful tool in the study of chemical structures with antioxidant activity. The highest occupied molecular orbital (HOMO), lowest unoccupied molecular orbital (LUMO), the ionization potential (I), and electronic affinity (A) are helpful to understand the molecular stability linked with the ability to accept or donate electrons [22,23,24]. The energy values of HOMO, LUMO, and I have also been associated with the amine groups’ reactivity and toxicity of DDS. Therefore, the amine group is the highest nucleophilic site and susceptible to oxidization [25]. These parameters will allow the generation of the donor-acceptor map (DAM), a useful tool to categorize any potential antioxidant substance as an electron donor or acceptor that permits us to better understand the possible antioxidant mechanism [26].

This work aims to: (i) Obtain new aromatic imine derivatives of DDS with aromatic aldehydes substituted in position 4, in order to evaluate their in vitro antioxidant activity through a chemical method using two experimental methodologies; and (ii) establish their correlationship with electronic descriptors of DFT calculations.

The selection of 4-substituted derivatives in this research project was based on literature reports where the antimicrobial and antioxidant activity of imine-type derivatives have a greater effect if the substituents occupy that position [27,28]. Furthermore, our study was directed in a complementary way to evaluate the influence of the type of electron-withdrawing/electron-attracting substituents in that position in DDS derivatives.

## 2. Results

### 2.1. Imine Synthesis

**Figure 1:**
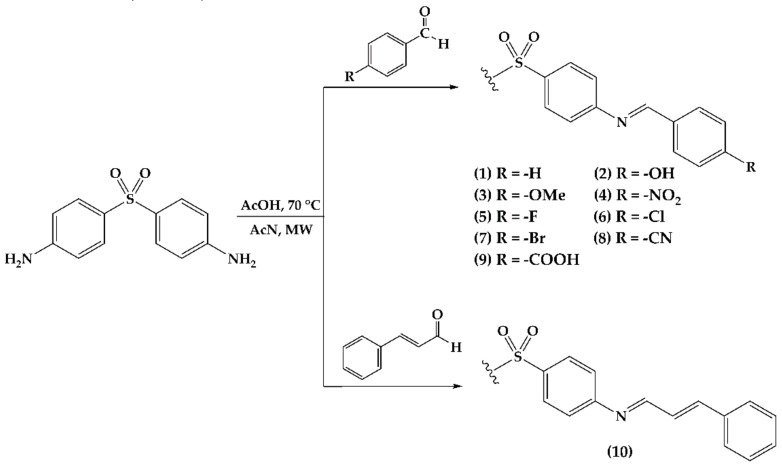
Reagents and conditions for DDS imine derivatives synthesis. Derivatives **2**, **3**, **5**, **7**, **8**, and **10** were obtained as monosubstituted compounds. Derivatives **1**, **4**, **6**, and **9** were obtained as *N*,*N*′-substituted compounds.

Ten imine derivatives **1**–**10** were obtained through condensation between DDS and the corresponding aromatic aldehydes 4-substuited (**1**–**9**) and cinnamaldehyde (**10**), using acetonitrile as the solvent (Figure 1), with reaction times between 3 to 7 h under microwave irradiation. All of the compounds were obtained as solids with yields ranging from 70–90% (Table 1). The derivatives’ structures were confirmed by nuclear magnetic resonance (NMR). The spectra showed the characteristic signal for imine proton at 8–9 ppm, and a doublet of doublets at the aromatic region for aldehyde protons.

### 2.2. Antioxidant Effects

The antioxidant activity of the synthesized compounds **1**–**10**, as well as the ascorbic acid and butylated hydroxytoluene (BHT) standards, were evaluated using two assays: The DPPH capture method (antiradical) [29] and the ferric reducing/antioxidant power assay (FRAP) [30].

#### 2.2.1. DPPH Radical Scavenging Assay

The DPPH radical capturing method is based on the measurement of light absorption at 517 nm (purple) that decreases when a radical reacts against an antioxidant agent, resulting in the reactive solution discoloration. BHT is used as an antioxidant reference, which is considered a lipophilic compound due to its preferred solubility on organic solvents and oils [31]. The mechanism is based on the ability of the hydroxy group to transform into a radical species that is highly stabilized by electronic delocalization on the aromatic ring [32].

As shown in Table 2, it was found that bulky halogens (**6** and **7**) and the nitro substituent (**4**) are incapable of capturing and stabilizing DPPH free radicals. The fluor (**5**) and cyano (**8**) substituents behave as well as the non-substituted ring (**1**), although the antioxidant effect remains lower than DDS. On the other hand, the hydroxy (**2**), methoxy (**3**), and 2-phenylethylen (**10**) groups have an incremented antioxidant effect, although the carboxylate group (**9**) has 26 times higher antioxidant activity than DDS. The antioxidant effect of **9** is 73%, similar to BHT. The tendency observed for radical capturing is **4**, **6** < **7** < **1** < **8** < **5** < DDS < **3** < **10** < **2** < **9**.

#### 2.2.2. Ferric Reducing/Antioxidant Power Assay (FRAP)

The reducing effect method is based on iron (III) reduction to iron (II) starting from a yellow ferricyanide complex solution. The reaction solution turns greenish-blue as the ferro-cyanide complex arises and can be measured at 750 nm. An antioxidant agent can promote the reduction reaction as AA and is capable of transferring two protons and two electrons to form the dehydroascorbic acid [33].

Using AA as a reference, DDS has a reducing effect of 17.3%. Derivatives **2**, **7**, and **8** have a similar activity as DDS (Table 3). Meanwhile, derivatives **1**, **5**, and **6** have a reducing effect in the range of 13.2–15.0%, representing the diminished activity with respect to the parent drug. Finally, derivatives **3**, **4**, **9**, and **10** have a substantial activity increment, reaching values 1.6 and 2.5 times higher than DDS.

### 2.3. DFT Analysis

The determination of the electronic parameters of a molecule is an important tool for interpreting and explaining its reactivity in antioxidative processes [34]. The frontier molecular orbitals HOMO and LUMO are descriptors related to the electron donation and electron acceptor behavior of a molecule, respectively. Additionally, the Gap_LUMO-HOMO_ value (Gap_LUMO-HOMO_ = E_LUMO_ − E_HOMO_) is related to the intramolecular charge transfer and it is indicative of the molecular chemical stability. The lower the Gap_HOMO-LUMO_, the higher the molecular reactivity and, therefore, the lower the chemical stability [35]. The calculated HOMO, LUMO, and Gap_HOMO-LUMO_ are shown in Table 4. The Gap_HOMO-LUMO_ values for all of the derivatives were lower than DDS pointing out to a general improvement of reactivity and following the tendency **8** < **10** < **4** < **7** < **9** < **5** < **3** < **2** < **6** < **1** < DDS. Consequently, it is expected that derivatives **4**, **7**, **8**, and **10** achieve the best reactivity values, while **2**, **6**, and **1** are similar to DDS.

Additionally, the values of the I and A descriptors, electrodonating (ω^−^), and eletro-accepting (ω^+^) capacities were calculated from the conceptual density functional theory (for details, see Section 4.4.1) [26,36,37,38,39,40,41,42]. The calculated I values showed the following sequence **3** < DDS < **10** < **2** < **5** < **7** < **8** < **1** < **6** < **9** < **4**. Additionally, the A values presented the sequence DDS < **3** < **2** < **5** < **1** < **7** < **10** < **6** < **9** < **8** < **4**, where the electron withdrawal compounds (**4**, **8**, and **9**) have a greater capacity to stabilize extra electrons (Table 5).

**Figure 2:**
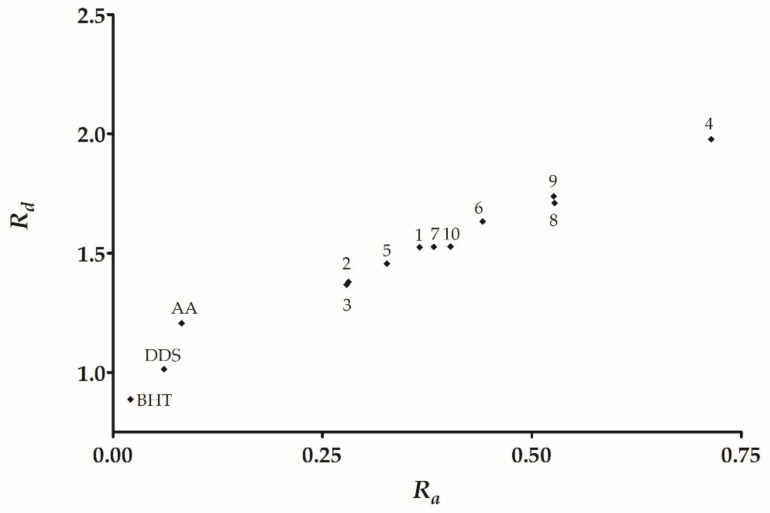
DAM for DDS derivatives **1**–**10**. The break is intended to visualize DDS in the DAM.

From the ω^−^ and ω^+^ and using Equations (6) and (7) (see Section 4.4.1), we calculated *R_a_* and *R_d_* to build the DAM [26,43], as shown in Figure 2. The DAM is useful for ranking a given compound in terms of its ability to accept or donate electrons. Comparing the studied derivatives, we found that derivatives **4**, **8**, and **9** showed the higher *R*_a_ and *R_d_* values. Therefore, they have the better capacity to accept a charge, acting as the best antireductant/antiradical derivatives. On the other hand, derivatives **2**, **3**, and **5** showed the lower *R_a_* and *R_d_* values labelling them as good antioxidants (electron donator species), with DDS being the best antioxidant.

## 3. Discussion

We prepared and characterized a DDS imine derivative series to assess their antioxidant potential compared to the parental drug. This compound series was evaluated by two in vitro models: (i) DPPH radical capturing; and (ii) ferric reducing antioxidant power. Our experimental findings were complemented with the DFT analysis in terms of (i) electronic distribution; (ii) the total charge accepting/donating capacity (I/A); (iii) the partial charge accepting/donating capacity (ω^+^/ω^−^); and (iv) an analysis of the relative donor-acceptor capacity through the DAM.

From the DPPH model, we found three trends: (a) Derivatives with null or lower activity than DDS, this set of derivatives have electron withdrawal groups (halogen **5**–**7**, cya-no **8**, and hydrogen **1**); (b) derivatives with an equivalent activity to DDS, hydroxy **2**, and cinnamyl **10**; and (c) derivatives with higher activity than DDS, specifically derivative **9** containing a carboxyl group. Therefore, the following tendency is observed carboxyl >> hydroxyl > cinnamyl > methoxy. Additionally, the reducing antioxidant/power showed a similar tendency, with the derivatives being minor or of similar activity to DDS that contain electron withdrawal groups. Moreover, the higher reducing power derivatives are carboxyl > nitro > cinnamyl > methoxy.

**Figure 3:**
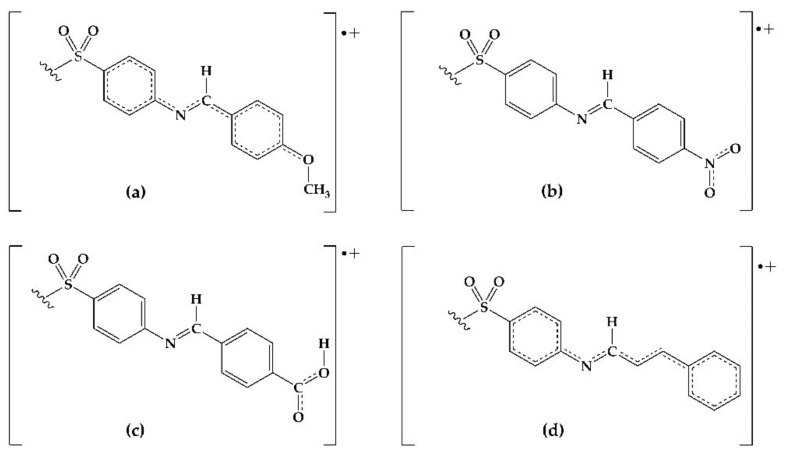
Possible resonant structures of derivatives (**a**) **2**, (**b**) **4**, (**c**) **9**, and (**d**) **10**.

The antioxidant phenomena observed can be explained by an electronic behavior analysis. For derivatives **2**, **3**, and **10**, the substituents act as electron donators (good antiradical agents) due to the induction or hyperconjugation that makes it capable of stabilizing the cation-radical species formed in the electron-donating process towards DPPH or Fe^3+^ (Figure 3). Derivative **9** is also capable of stabilizing the cation-radical due to the electronic resonance on the carboxyl group. In the case of **4**, the resonance by the nitro group explains its effectivity in the reducing power model. On the contrary, those derivatives with electron-withdrawing groups (F, Cl, Br, CN) decrease the electronic transfer availability to DPPH radical. Consequently, a null or minor activity with respect to DDS is observed.

The calculation of molecular electronic parameters is relevant to predict the reactivity in several chemical reactions. The frontier molecular orbitals (HOMO and LUMO) are two crucial descriptors since they represent the electron-donating and electron-withdrawing capacities of a given molecule. Comparing the HOMO values of DDS and its derivatives (Table 4), we observed that all of the compounds presented lower values than DDS. Among the derivative series, **2**, **3**, and **10** have higher HOMO values that can be understood as a greater facility towards the electron donation, explaining the suitable antioxidant activities observed in vitro. On the other hand, according to the Gap_HOMO-LUMO_, derivatives **4**, **8**, and **10** have smaller values that involve a higher reactivity correlated to the antioxidant behavior in reducing the power assay (Table 3).

**Figure 4:**
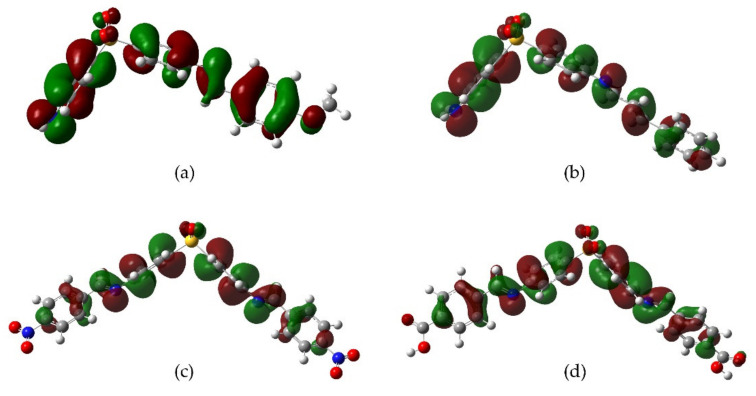
HOMO distribution for the most active derivatives (**a**) methoxy **3**, (**b**) cinnamoyl **10**, (**c**) nitro **4**, and (**d**) carboxyl **9**.

Derivatives **3**, **4**, and **10** have a homogeneous HOMO distribution along with their molecular structure, while **9** showed the distribution mainly over the aromatic rings of DDS moiety. On the other hand, the LUMO distribution is predominantly localized in the aromatic rings linked by the imine group, this behavior is notable for derivative **4**. These observations led us to propose different scenarios where an unbalanced distribution of orbitals is required to produce the antioxidant effect (Figure 4).

The low I values represent compounds that are easily oxidizable and act as efficient antiradical agents depending on their specific electro-donative capacity. For example, in Table 5, we found that derivatives **2**, **3**, **5**, and **10** have the lowest I value that can be understood as a higher ease to transfer one electron and, therefore have better antiradical activity. In opposition, derivatives **1**, **4**, **6**, and **9** have the highest I value in the series, explaining the low DPPH radical capturing activity of these compounds, except for compound **9**.

The results for A showed that all of the derivatives have positive values, while the DDS value is negative. The positive values indicate that the anionic structure is more stable than its corresponding neutral form. Therefore, they have a greater capacity for accepting electrons than DDS. Consequently, DDS is a less efficient antireductant. Among the derivatives, the most effective ones as antireductants are **4** > **8** > **9**, and the lowest effective ones are **3** < **2** < **5**. This interpretation of A allows us to explain that derivative **9** showed the highest activities in the DPPH radical capturing assay, based on a reaction mechanism that involves the interaction of our compound with the radical species, the electron capture, and stabilization through electronic delocalization.

Highly effective electron donors have lower values of electron-donating capacity (ω). All of the derivatives have higher values than DDS, particularly, derivatives **2**, **3**, and **5** have the lowest values within the series. With respect to the I values, it is expected that a low I value implies a tendency towards electronic transfer/donation. However, the I value for our derivatives, particularly, derivatives **4**, **6**, and **9** are higher than DDS. Consequently, a reactivity trend referring to ω^−^ is proposed, as follows: BHT < DDS < AA < **3** < **2** < **5** < **1** < **7** < **10** < **6** < **8** < **9** < **4**. For the electro-accepting capacity (ω^+^), high values are related to an effective electronic acceptor species. In our study, the trend for ω^+^ is the following: BHT < DDS < AA < **3** < **2** < **5** < **1** < **7** < **10** < **6** < **9** < **8** < **4**. We observed here a DDS as the lowest electron acceptor in the trend above. This result correlates with the A values where the DDS has a negative value, indicating an antioxidant behavior (good electron donator). The highest ω^+^ values were obtained for the derivative series for **4**, **8**, and **9** (good antireductant activity), and their experimental activity was corroborated in ferric reducing the antioxidant test. On the other hand, derivative **3** has the lowest ω^+^ and showed an antioxidant activity in the ferric reducing antioxidant test.

The DAM allows us to classify substances based on their electron-donating or accepting capacity [26,43]. This work showed that DDS is positioned below BHT and AA (Figure 2). Therefore, DDS has a lower antioxidant activity than the control compound BHT. Furthermore, all of the derivatives have *R_a_* and *R_d_* values higher than DDS. Therefore, the full derivative series has a poor electron-donating capacity, but a good electron-accepting capacity with respect to DDS.

We found that derivatives **2** and **3** have the lowest *R_a_* values representing a good antioxidant behavior that is observed experimentally in DPPH radical capturing. However, **9** has an outstanding experimental result, followed by **2**, **3**, and **10**. Regarding *R_d_*, derivatives **4** and **9** have the highest values, and thus they classify as the compound with the better electron-withdrawing capacity into the derivative series. Furthermore, derivatives **4** and **9** display the best-reducing capacity activity in addition to the highest ω^+^ and *R_d_*. Consequently, we propose these as the best antireductants in our DDS imine derivative series.

The antioxidant property of a specific compound is of interest due to its possible scavenging of active radicals that could trigger pathologies as cancer. The proposed derivatives were evaluated by in silico methodologies according to their possible cytotoxic activity, using the CLC-pred tool (Table S1) [44]. It was found that DDS is an active compound on the ovarian carcinoma cell line (Pa = 0.625), while derivatives **1**–**9** showed an activity on colon adenocarcinoma. Derivatives **1**, **4**, and **6** are the best evaluated values with Pa = 0.681 (**1**), Pa = 0.539 (**4**), and Pa = 0.572 (**6**). Derivative **10** is an active compound on pancreatic carcinoma, even though it has a low value (Pa = 0.391).

Otherwise, the in silico acute-oral toxicological profile of the synthesized compounds was determined using the SiliS-PTOXRA software [45]. In general, it was found that the compounds are within category III of the Environmental Protection Agency (EPA) and in categories IV and V of the Globally Harmonized System (GHS), suggesting that these compounds have low oral toxicity (Table S2).

## 4. Materials and Methods

### 4.1. Materials and Equipment

DDS (CAS 80-08-0), benzaldehyde (CAS 100-52-7), 4-hydroxybenzaldehyde (CAS 123-08-0), 4-methoxybenzaldehyde (CAS 123-11-5), 4-fluorobenzaldehyde (CAS 459-57-4), 4-chlorobenzaldehyde (CAS 104-88-1), 4-bromobenzaldehyde (CAS 1122-91-4), 4-nitrobenzaldehyde (CAS 555-16-8), 4-formylbenzonitrile (CAS 105-07-7), 4-formylbenzoic acid (CAS 619-66-9), cinnamaldehyde (CAS 104-55-2), butylated hydroxytoluene (BHT, CAS 128-37-0), and glacial acetic acid (CAS 64-19-7) were purchased from Sigma-Aldrich^®^ (Ciudad de Mexico, Mexico), and used without prior purification. Ascorbic acid (CAS 50-81-7), potassium ferricyanide (CAS 13746-66-2), sodium dodecyl sulfate (SDS, CAS 151-21-3), ferric chloride (FeCl_3_.6H_2_O, CAS 7705-08-0), hydrochloric acid (CAS 7647-01-0), DPPH free radical (CAS 1898-66-4), and all of the solvents used were grade ACS and purchased from J. T. Baker^®^ (Ciudad de Mexico, Mexico). The solvents were dried and purified in agreement with standard procedures [46]. The microwave-assisted synthesis (MWAS) was performed in a CEM^®^ Discovery BenchMate apparatus (CEM Corporation, Matthews, NC, USA). The reactions were monitored by thin-layer chromatography (TLC) on Merk^®^ silica gel 60 F_254_ plates. Iodine vapour was used as a detecting agent. The melting points were measured in a Dynalon Afon™ DMP100 apparatus and reported without correction. NMR spectra were recorded in a 300–600 MHz Bruker^®^ Advance III using DMSO-d_6_ or CDCl_3_ as a solvent. The absorbance measurements were performed in a Perkin Elmer^®^ Lambda XLS-plus spectrophotometer using quartz cells with a 1 cm path length.

### 4.2. DDS Imine Derivative Synthesis

The synthesis employed herein is based on a modification of Wadher’s procedure [20]. Three milliliters of acetonitrile was poured into a 10 mL round flask provided with a magnetic stirrer. Then, DDS (1.0 mmol) and the aldehyde (1.25 mmol) were dissolved. Thereafter, a small aliquot of glacial acetic acid is added as a catalyzer. The mixture was irradiated (MWAS) at 70 °C and a potency of 100 watts in intervals of 30 min. The reaction is monitored by TLC (hexane:ethyl acetate 4:6) until the total disappearance of the starting materials. The crude was rinsed and precipitated with hexane, methanol, and acetone (see Figure 1 and Table 1).

(1*E*,1′*E*)-*N*,*N*′-(sulfonylbis(4,1-phenylene))bis(1-phenylmethanimine) (**1**). Amorphous white solid with 90% yield; m.p. 213.0–214.6 °C. ^1^H-NMR (CDCl_3_, 400 MHz); δ: 8.38 (s, 2H), 7.97 (ddd, *J* = 8.7, 2.3 Hz, 4H), 7.91–7.85 (m, 4H), 7.52–7.44 (m, 6H), 7.25 (ddd, *J* = 8.6, 1.7 Hz, 4H). ^13^C-NMR: 162.7, 156.5, 135.5, 129.2, 121.5, 138.5, 128.9, 132.2.

(*E*)-4-(((4-((4-aminophenyl)sulfonyl)phenyl)imino)methyl)phenol (**2**). Amorphous yellow solid with 70% yield; m.p. 129.8–130.9 °C. ^1^H-NMR (DMSO-*d*_6_, 400 MHz); δ: 9.90 (s, 1H, OH), 8.43 (s, 1H), 7.82 (ddd, *J* = 8.6, 2.3, 2.3 Hz, 2H), 7.77 (ddd, *J* = 8.5, 2.7, 1.9 Hz, 2H), 7.56 (ddd, *J* = 8.7, 2.6, 2.0 Hz, 2H), 7.29 (ddd, *J* = 8.4, 2.7, 1.8 Hz, 2H), 6.90 (ddd, *J* = 8.5, 2.7, 1.4 Hz, 2H), 6.60 (ddd, *J* = 8.5, 2.4, 1.9 Hz, 2H) y 6.00 (s, 2H). ^13^C-NMR: 162.6, 161.7, 156.2, 153.9, 131.6, 129.7, 128.9, 128.3, 122.0, 116.3; 113.6.

(*E*)-4-((4-((4-methoxybenzylidene)amino)phenyl)sulfonyl)aniline (**3**). Amorphous white powder with 90% yield; m.p. 227.0–228.2 °C. ^1^H-NMR (DMSO-*d*_6_, 600 MHz,); δ: 8.46 (s, 1H), 7.84 (d, *J* = 8.8 Hz, 2H), 7.80 (d, *J* = 8.6 Hz, 2H), 7.53 (d, *J* = 8.8 Hz, 2H), 7.28 (d, *J* = 8.6 Hz, 2H), 7.03 (d, *J* = 8.8 Hz, 2H), 6.59 (d, *J* = 8.8 Hz, 2H), 6.14 (s, 2H), 3.79 (s, 3H). ^13^C-NMR: 163.9, 162.8, 156.0, 154.1, 140.2, 131.5, 129.9, 129.0, 128.4, 126.3, 122.2, 114.9, 113.6, 56.0.

(1*E*,1′*E*)-*N*,*N*′-(sulfonylbis(4,1-phenylene))bis(1-(4-nitrophenyl)methanimine) (**4**). Amorphous yellow solid with 81% yield; m.p. 234.0–236.0 °C. ^1^H-NMR (DMSO-*d*_6_, 300 MHz); δ: 8.80 (s, 2H), 8.38 (dd, *J* = 8.9, 2.1 Hz, 4H), 8.19 (dd, *J* = 9.0, 2.0 Hz, 4H), 8.06 (d, *J* = 8.6 Hz, 2H), 7.51 (d, *J* = 8.6 Hz, 2H). ^13^C-NMR: 162.2, 155.3, 152.8, 149.3, 140.8, 138.7, 130.2, 128.9, 128.6, 128.1, 124.1, 122.3.

(*E*)-4-((4-((4-fluorobenzylidene)amino)phenyl)sulfonyl)aniline (**5**). Amorphous white solid with 81% yield; m.p. 163.1–164.3 °C. ^1^H-NMR (DMSO-*d*_6_, 600 MHz,); δ: 8.59 (s, 1H), 8.00 (m, 4H), 7.86 (d, *J* = 8.5 Hz, 2H), 7.44 (m, 2H), 7.36 (m, 2H), 6.63 (d, *J* = 8.8 Hz, 2H), 6.19 (s, 2H). ^13^C-NMR: 162.5, 155.5, 154.1, 140.7, 132.9, 132.0, 130.0, 129.3, 129.1, 128.4, 122.3, 116.5, 113.3.

(1*E*,1′*E*)-*N*,*N*′-(sulfonylbis(4,1-phenylene))bis(1-(4-chlorophenyl)methanimine) (**6**). Amorphous white solid with 90% yield; m.p. 204.5–205.3 °C. ^1^H-NMR (CDCl_3_, 400 Hz); δ: 8.35 (s, 1H), 8.34 (s, 1H), 7.97 (d, 2H), 7.90 (d, 2H), 7.82 (dd, 4H), 7.48 (dd, 4H), 7.24 (d, 2H), 7.19 (d, 2H). ^13^C-NMR: 161.0, 160.0, 156.2, 155.6, 138.7, 138.4, 138.0, 134.0, 130.4, 130.1, 129.2, 129.0, 128.5, 121.5, 121.3.

(*E*)-4-((4-((4-bromobenzylidene)amino)phenyl)sulfonyl)aniline (**7**). Amorphous yellow solid with 75% yield; m.p. 191.7–194.1 °C. ^1^H-NMR (DMSO-*d*_6_, 600 MHz); δ: 8.55 (s, 1H), 7.83 (m, 4H), 7.70 (m, 2H), 7.53 (d, *J* = 8.8 Hz, 2H), 7.33 (d, *J* = 8.6, 2H), 6.59 (d, *J* = 8.6, 2H), 6.15 (s, 2H). ^13^C-NMR: 162.7, 155.3, 154.1, 140.8, 135.2, 132.5, 131.4, 131.3, 129.9, 129.3, 128.43, 122.3, 113.6.

(*E*)-4-(((4-((4-aminophenyl)sulfonyl)phenyl)imino)methyl)benzonitrile (**8**). Amorphous white powder with 75% yield; m.p. 123.9–124.7 °C. ^1^H-NMR (DMSO-*d*_6_, 600 MHz); δ: 8.67 (s, 1H), 8.06 (m, 2H), 7.97 (m, 2H), 7.85 (m, 2H), 7.53 (d, *J* = 9.0 Hz, 2H), 7.38 (d, *J* = 8.7 Hz, 2H), 6.59 (d, *J* = 9.0 Hz, 2H), 6.16 (s, 2H). ^13^C-NMR: 167.7, 155.9, 154.9, 141.3, 139.3, 133.0, 130.1, 129.3, 128.2, 122.5, 119.0, 116.7, 113.6.

(*E*)-4-(((4-((4-aminophenyl)sulfonyl)phenyl)imino)methyl)benzoic acid (**9**). Amorphous white solid with 77% yield; m.p. 388 °C (dec.) ^1^H-NMR (DMSO-*d*_6_, 600 MHz); δ: 8.66 (s, 1H), 8.65 (s, 1H), 8.04 (dd, *J* = 8.2, 3.5 Hz, 4H), 7.84 (m, 4H), 7.41 (td, *J* = 8.7, 1.7 Hz 4H), 7.36 (m, 4H). ^13^C-NMR: 167.1, 163.0, 156.2, 153.3, 139.4, 136.2, 129.7, 128.4, 122.3, 113.6.

4-((4-(((1*E*,2*E*)-3-phenylallylidene)amino)phenyl)sulfonyl)aniline (**10**). Amorphous yellow solid with 71% yield; m.p. 212.4–213.5 °C. ^1^H-NMR (DMSO-*d*_6_, 600 MHz); δ: 8.32 (d, *J* = 9.0 Hz, 1H), 7.80 (d, *J* = 8.6 Hz, 2H), 7.65 (d, *J* = 7.0 Hz, 2H), 7.53 (d, *J* = 8.8 Hz, 2H), 7.42–7.34 (m, 4H), 7.25 (d, *J* = 8.6 Hz, 2H), 7.14 (dd, *J* = 16.0, 9.0 Hz, 1H), 6.59 (d, *J* = 8.8 Hz, 2H), 6.15 (s, 2H). ^13^C-NMR 165.0, 155.6, 154.1, 146.5, 140.5, 135.7, 129.9, 129.5, 128.6, 128.4, 128.4, 128.4, 126.2, 122.1, 113.6.

### 4.3. Antioxidant Activity

All of the DDS derivatives were evaluated as potential antioxidant agents using the DPPH radical capturing method [29] and the ferric reducing antioxidant power [30].

#### 4.3.1. DPPH Radical Scavenging Assay

In a glass assay tube, 2 mL of a methanolic solution of antioxidant agent (250 μg/mL, 0.5–1.0 mM) was mixed with 2 mL of a methanolic solution of DPPH (20 μg/mL, 0.05 mM) or the control, which is butylated hydroxytoluene (BHT) (10 μg/mL, 0.05 mM). The solution was incubated at RT for 60 min. Then, the absorbance was measured at 517 nm. The results are expressed as a percentage with respect to the DPPH reference solution. The neutralization percentage was calculated following the formula:

(1) $$\%\mathrm{Capture}=\frac{\mathrm{Ac}-\mathrm{As}}{\mathrm{Ac}}\times 100$$

where Ac is the absorbance of the DPPH reference solution and As is the absorbance of the sample.

#### 4.3.2. Ferric Reducing Antioxidant Power

The following mixture was prepared in a glass assay tube: 0.5 mL of the antioxidant agent (250 µg/mL, 0.5–1.0 mM), 0.75 mL of HCl 1.0 M, 0.75 mL of ferricyanide (1% *w*/*v*), 0.25 mL of sodium dodecyl sulfate (1% *w*/*v*), and 0.25 mL of FeCl_3_·6H_2_O (0.2% *w*/*v*). The mixture was incubated at 50 °C on a water bath for 30 min, cooled to RT, and the absorbance was measured at 750 nm against a reagent blank. Finally, the mixture was compared against an ascorbic acid positive control solution (10 µg/mL, 0.06 mM). The results are expressed as a percentage with respect to the ascorbic acid (AA) control.

#### 4.3.3. Statistical Data Analysis

The statistical analysis was performed using GraphPad Prism^®^ 5. For both antioxidant assays, the data are presented as the average ± SD and each trial was performed in triplicate as an independent experiment. These values were compared by ANOVA, followed by the Dunnett test. Values of *p* < 0.05 are considered as meaningful.

### 4.4. Computational Details

Electronic structure calculations were carried out using the density functional theory (DFT), at CAM-B3LYP/6-311G (d,p/level of theory [47], implemented in the Gaussian09 software. All of the calculations were performed in a vacuum. The thermodynamic values for zero-point vibrational energy (ZPVE), the thermal energy, and the absolute entropies were obtained by frequency calculations for each minimum energy point. This type of calculation was performed considering an ideal behavior from the harmonic frequencies and the standard moments of inertia at 1 atm and 298 K for pressure and temperature, respectively. The minimum energy structures were verified using frequency calculations, and all of the structures presented an entire database of positive real values (NImag = 0) [48]. The minimum energy structures were used to obtain the electronic and molecular descriptors, HOMO and LUMO [49].

#### 4.4.1. Electronic and Topological Parameters

The Gibbs enthalpy, entropy, and free energy values for the Schiff base studied were obtained from vibrational calculations. The vertical ionization potential (I) and vertical electronic affinity (A) were calculated from the total energy of optimized structures as neutral (E_Neutral_), anionic (E_Anion_), and cationic (E_Cation_) species with the following equations [36,37]:

I = E_Cation_ − E_Neutral_ (2)

A = E_Neutral_ − E_Anion_ (3)

All of the electronic descriptors were obtained from the conceptual density functional theory [38,39,40]. The I and A parameters allowed us to bring two parameters related to the charge transfer process: The electro-donating (ω^−^, Equation (4)) and electro-accepting (ω^+^, Equation (5)) capacities. These capacities have a parallel behavior with respect to I and A, although the interpretation is different since I and A measure the capacity of a chemical species to donate or accept one electron, while ω^−^ and ω^+^ measure the capacity to donate or accept a small fractional amount of charge. It is essential to mention that the higher the ω^+^ value, the higher the capability to accept a charge, and the lower the ω^−^ value, the higher the capacity to donate a charge [41,42].

(4) $$\omega^{-}=\frac{{\left(3I+A\right)}^{2}}{16\;\left(I-A\right)}$$

(5) $$\omega^{+}=\frac{{\left(I+3A\right)}^{2}}{16\;\left(I-A\right)}$$

**Figure 5:**
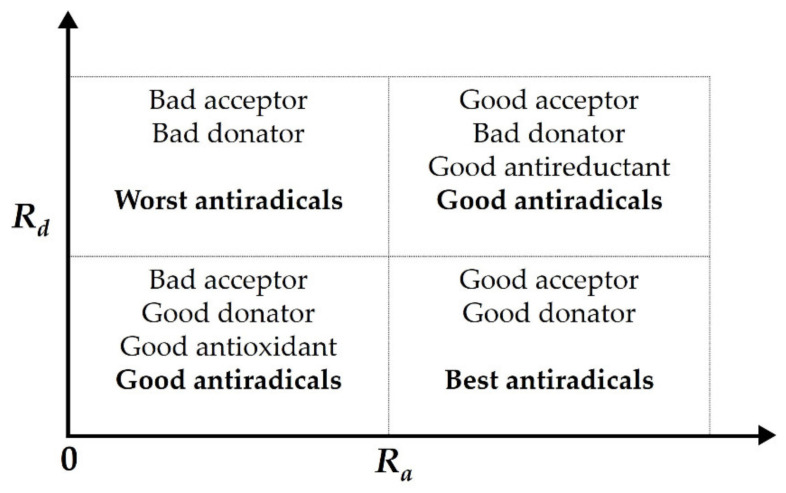
Donor-acceptor map (DAM). Adapted from [26,43].

Finally, the electron acceptation index (*R_a_*, Equation (6)) and the electron donation index (*R_d_*, Equation (7)) were calculated. F and Na were used as models for suitable electron acceptors and good electron donors, as described by Martinez [26,43].

(6) $$R_{a}=\frac{\omega_{L}^{+}}{\omega_{F}^{+}}$$

(7) $$R_{d}=\frac{\omega_{L}^{-}}{\omega_{Na}^{-}}$$

where ω*_L_* is the value associated with the compound of interest, while ω*_F_* and ω*_Na_* are the values obtained for F and Na, respectively. *R_a_* and *R_d_* can take values higher or lower than 1 in four combinations, as represented in the donor-acceptor map (Figure 5). Therefore, any antioxidant substance with known *R_a_* and *R_d_* indexes is susceptible to be described in terms of the DAM.

## 5. Conclusions

In the present work, the antioxidant activity of 10 dapsone derivatives was evaluated by two in vitro models (DPPH radical scavenging assay and ferric reducing antioxidant power). To explain our experimental data, DFT calculations were performed and the relative donor-acceptor capacity was analyzed through the DAM. The derivatives that showed the best results, according to their antioxidant activity in both in vitro and in silico tests, were **2**, **3**, **4**, **9**, and **10**. The potential use of these derivatives as anticancer agents was evaluated in silico, considering that one of the conditions for the development of this pathology is the oxidative stress generated by free radicals. All of the derivatives showed a potential activity as anticancer agents, particularly **1**, **4**, and **6** presented Pa > 0.5 values related to colon carcinoma. According to the toxicity analysis, all our DDS derivatives showed low values in accordance with the EPA and GHS categories. Due to the results found in this work, in the future our derivatives, particularly **4** and **9**, may have applications in the medical area as potential therapeutic agents, specifically in processes where oxidative stress is involved.

## Figures and Tables

**Table 1:** Summary of DDS imine derivatives synthesis.

Derivative	Substituent (R)	Time (h)	%Yield	Melting Point °C
**1**	Hydrogen	2	90	213.0–214.6
**2**	4-hydroxyl	3	70	129.8–130.9
**3**	4-methoxy	3	90	227.0–228.2
**4**	4-nitro	3	81	234.0–236.0
**5**	4-fluoro	5	81	163.1–164.3
**6**	4-chloro	3	90	204.5–205.3
**7**	4-bromo	7	75	191.7–194.1
**8**	4-cyano	3	75	123.9–124.7
**9**	4-carboxylate	3	77	388 (decomp.)
**10**	2-phenylethylen	3	71	212.4–213.5

**Table 2:** Percentage of DPPH radical capturing and correlation ratios against DDS and BHT.

Derivative	Substituent	%Capture	Ratio Derivative/DSS	Ratio Derivative/BHT
**1**	Hydrogen	1.5 ± 1.2 ns	0.60	0.017
**2**	4-hydroxyl	5.8 ± 0.6 **	1.92	0.064
**3**	4-methoxy	3.3 ± 1.9 ns	1.32	0.036
**4**	4-nitro	0.0 ± 1.8 ns	0.00	0.000
**5**	4-fluoro	1.7 ± 0.4 ns	0.68	0.019
**6**	4-chloro	0.0 ± 1.2 *	0.00	0.000
**7**	4-bromo	0.1 ± 0.5 *	0.04	0.001
**8**	4-cyano	1.6 ± 1.6 ns	0.64	0.018
**9**	4-carboxyl	66.2 ± 0.5 ***	26.48	0.730
**10**	2-phenylethylen	4.0 ± 0.8 ns	1.60	0.044
DDS		2.5 ± 0.3	1.00	0.028
BHT		90.7 ± 0.3	36.28	1.00

* Significative (*p* < 0.05); ** (*p* < 0.01); *** (*p* < 0.001); ns: Non significative. Note: All of the data are expressed as average ± SD in triplicate as independent experiments.

**Table 3:** Percentage of the reducing effect and correlation with DDS and ascorbic acid.

Compound	Substituent	%Reduction	Ratio Derivative/DDS	Ratio Derivative/AA
**1**	Hydrogen	15.0 ± 0.7 **	0.9	0.15
**2**	4-hydroxyl	16.9 ± 1.3 ^ns^	1.0	0.17
**3**	4-methoxy	28.4 ± 0.8 ***	1.6	0.28
**4**	4-nitro	40.7 ± 0.2 ***	2.4	0.41
**5**	4-fluoro	13.2 ± 0.3 ***	0.8	0.13
**6**	4-chloro	14.5 ± 0.4 ***	0.8	0.15
**7**	4-bromo	19.3 ± 1.4 **	1.1	0.19
**8**	4-cyano	20.0 ± 0.8 ***	1.2	0.20
**9**	4-carboxyl	44.8 ± 0.7 ***	2.6	0.45
**10**	2-phenylethylen	39.6 ± 0.1 ***	2.3	0.40
DDS		17.3 ± 0.6	1.0	0.17
AA		100.0 ± 0.3	5.8	1.00

* Significative (*p* < 0.05); ** (*p* < 0.01); *** (*p* < 0.001); ^ns^: Non significative. Note: All of the data are expressed as average ± SD in triplicate as independent experiments.

**Table 4:** HOMO, LUMO, and Gap_HOMO-LUMO_ energy values.

Derivative	HOMO (eV)	LUMO (eV)	Gap_HOMO-LUMO_ (eV)
**1**	−7.82	−0.99	6.83
**2**	−7.42	−0.69	6.72
**3**	−7.37	−1.93	6.70
**4**	−8.33	−2.03	6.31
**5**	−7.52	−0.89	6.63
**6**	−7.96	−1.23	6.73
**7**	−7.54	−1.05	6.49
**8**	−7.66	−1.57	6.09
**9**	−8.05	−1.50	6.55
**10**	−7.43	−1.15	6.28
**DDS**	−7.25	−0.30	6.95
**BHT**	−7.17	1.23	8.40
**AA**	−8.22	0.42	8.64

**Table 5:** Molecular descriptors calculated for DDS and its derivatives at a CAM-B3LYP theory level and 6-311G (d,p) basis set. Values in eV.

Compound	I	A	ω^−^	ω^+^	*R_a_*	*R_d_*
**1**	8.14	0.64	5.24	0.84	0.37	1.53
**2**	7.84	0.33	4.73	0.65	0.28	1.38
**3**	7.77	0.33	4.69	0.64	0.28	1.37
**4**	8.63	1.65	6.79	1.65	0.71	1.98
**5**	7.98	0.51	5.00	0.75	0.33	1.46
**6**	8.27	0.90	5.60	1.02	0.44	1.63
**7**	8.00	0.71	5.24	0.88	0.38	1.53
**8**	8.14	1.17	5.87	1.22	0.53	1.71
**9**	8.35	1.16	5.97	1.21	0.53	1.74
**10**	7.83	0.80	5.24	0.93	0.40	1.53
DDS	7.78	−1.10	3.48	0.14	0.06	1.01
BHT	7.63	−1.64	3.04	0.05	0.02	0.89
AA	9.06	−1.16	4.14	0.19	0.08	1.21
F	21.26	1.84	13.86	2.31	1.00	4.04
Na	5.38	0.40	3.43	0.54	0.23	1.00

## Supplementary Materials

The following are available. Table S1: In silico prediction of cytotoxicity for tumor cell lines. Table S2: Classification of the compounds in terms of the EPA and GSH categories for the evaluation of acute oral toxicity.
