# Supplementary material for: Synthesis, In Vitro, and In Silico Analysis of the Antioxidative Activity of Dapsone Imine Derivatives
Source: Molecules. 2021 Sep 22;26(19):5747. doi: 10.3390/molecules26195747 (PMC8510498; doi:10.3390/molecules26195747)
Supplement: Supplementary file 1 [file molecules-26-05747-s001.zip › molecules-1375140-supplementary.pdf]

Supplementary Material

Synthesis, *In Vitro*, and *In Silico* Analysis of the Antioxidative Activity of Dapsone Imine Derivatives †

Ricardo Guzmán-Ávila [1], Mayra Avelar [2], Edgar A. Márquez [3], Julio C. Rivera-Leyva [1], José R. Mora [4], Virginia Flores-Morales [2,*] and Jesús Rivera-Islas [1,*]

[1] Laboratorio 4, Facultad de Farmacia, Universidad Autónoma del Estado de Morelos, Av. Universidad 1001, 62209 Cuernavaca, Mexico; ricardo.guzman@uaem.edu.mx (R.G.-A.); julio.rivera@uaem.mx (J.C.R.-L.)

[2] Laboratorio de Síntesis Asimétrica y Bioenergética (LSAyB), Ingeniería Química (UACQ), Universidad Autónoma de Zacatecas, Campus XXI Km 6 Carr. Zac-Gdl, 98160 Zacatecas, Mexico; mayra.avelar@uaz.edu.mx

[3] Grupo de Investigación en Química y Biología, Departamento de Química y Biología, Universidad del Norte, Km 5 vía Puerto Colombia 1569, 081007 Barranquilla Atlántico, Colombia; ebrazon@uninorte.edu.co

[4] Grupo de Química Computacional y Teórica (QCT-USFQ), Departamento de Ingeniería Química, Universidad San Francisco de Quito, Diego de Robles y Vía Interoceánica, Quito 170901, Ecuador; jrmora@usfq.edu.ec

* Correspondence: virginia.flores@uaz.edu.mx (V.F.-M.); rij@uaem.mx (J.R.-I.); Tel.: +52-4929-256-690 (V.F.-M.); Tel.: +52-7773-2970-00 (J.R.-I.)

† Taking in part from the Ph. D. Thesis of Ricardo Guzmán-Ávila

DDS and its derivatives were subjected to their anticancer activity prediction through the CLC-pred server ((http://www.way2drug.com/Cell-line/index.php) [44]. For a given compound, this tool predicts the spectrum of cytotoxicity based on the structure-cytotoxicity relationship analysis for more than 50 000 active compounds on 278 human cancer cell lines. As can be observed in Table S1, the result is given as probability of activity (Pa) and probability of inactivity (Pi). For a compound, the probability to be active is considered when Pa > Pi. Otherwise, when Pa values > 0.5 a high probability to have anticarcinogenic activity is considered.

Table S1. *In silico* prediction of cytotoxicity for tumor cell lines.

Compound	Cell Line	Cell Line Full Name	Pa*	Pi**
**DDS**	A2780	Ovarian Carcinoma	0.625	0.005
**1**	LoVo	Colon adenocarcinoma	0.681	0.004
**2**	LoVo	Colon adenocarcinoma	0.473	0.006
**3**	LoVo	Colon adenocarcinoma	0.421	0.008
**4**	LoVo	Colon adenocarcinoma	0.539	0.005
**5**	LoVo	Colon adenocarcinoma	0.451	0.007
**6**	LoVo	Colon adenocarcinoma	0.572	0.005
**7**	LoVo	Colon adenocarcinoma	0.434	0.008
**8**	LoVo	Colon adenocarcinoma	0.534	0.005
**9**	LoVo	Colon adenocarcinoma	0.477	0.006
**10**	YAPC	Pancreatic carcinoma	0.391	0.112

*Pa (0 – 1) represents the bioactivity probability of a given compound based on structural similarity respect to an active molecule database. **Pi (0 – 1) represents the bioinactivity probability of a given compound based on structural similarity respect to an active molecule database.

In silico acute-oral toxicity

The acute-oral toxicity of the synthetized compounds was evaluated based on the Environmental Protection Agency (EPA) and Globally Harmonized System (GHS) categorization, which have been well accepted for the evaluation of the organic compound toxicity by considering the 50% Lethal Dose (LD_{50}) values. The prediction was performed by using the software SiliS-PTOXRA (http://tomocomd.com/apps/ptoxra) developed by the Marrero group [45]. As can be observed in Table S2, only one compound (**4**) was found to be out of the applicability domain and consequently its prediction was not determined. All other compounds were satisfactorily evaluated, and the results suggest that all of them are in class III of the EPA category, and class IV and V in the GHS category, suggesting that these compounds have, in general, low toxicity. This categorization was obtained for compounds with $300 < LD_{50} < 5000$ mol/kg.

Table S2. Classification of the compounds in terms of the EPA and GSH categories for the evaluation of acute oral toxicity.

		Acute-Oral Toxicity	
**Compound**	Applicability domain*	EPA category	GHS category
**1**	IN	III	V
**2**	IN	III	V
**3**	IN	III	IV
**4**	OUT	---	---
**5**	IN	III	IV
**6**	IN	III	V
**7**	IN	III	V
**8**	IN	III	IV
**9**	IN	III	V
**10**	IN	III	IV
**DDS**	IN	III	IV

*The labels "IN" and "OUT are used for those cases when the structures fall or do not fall within the model applicability domain, used for the prediction.
